# Tuberculosis risk factors among tuberculosis patients in Kampala, Uganda: implications for tuberculosis control

**DOI:** 10.1186/s12889-015-1376-3

**Published:** 2015-01-21

**Authors:** Bruce J Kirenga, Willy Ssengooba, Catherine Muwonge, Lydia Nakiyingi, Stephen Kyaligonza, Samuel Kasozi, Frank Mugabe, Martin Boeree, Moses Joloba, Alphonse Okwera

**Affiliations:** Department of Medicine, Makerere University College of Health Sciences, P.O. Box 7072, Kampala, Uganda; Department of Microbiology, Makerere University College of Health Sciences, Kampala, Uganda; National Tuberculosis and Leprosy Program, Ministry of Health, Kampala, Uganda; Department of Pulmonary Diseases, Radboud University Nijmegen Medical Center, Nijmegen, The Netherlands; National Tuberculosis treatment unit, Mulago Hospital, Kampala, Uganda; Department of Global Health and Amsterdam Institute of Global Health and Development, Academic Medical Center, University of Amsterdam, P.O. Box 22700, 1100 DE Amsterdam, Netherlands

## Abstract

**Background:**

Slow decline in the incidence of tuberculosis (TB) has been observed in most high TB burden countries. Knowledge of the prevalence of different TB risk factors can help expand TB control strategies. However with the exception of Human Immunodeficiency Virus (HIV) the prevalence of the other TB risk factors are poorly studied in Uganda. We aimed to determine the prevalence of different TB risk factors and TB disease presentation among TB patients in Kampala Uganda.

**Methods:**

We assessed 365 adult TB patients and used descriptive statistics to summarize their socio-demographic, clinical, radiological, sputum mycobacteriology and TB risk factors (HIV, diabetes, TB contact, alcohol use, tobacco smoking, poverty and overcrowding) data.

**Results:**

A total of 158 (43.3%) patients were male and the median age was 29 (IQR 28–30). Majority of the patients (89.2%) had pulmonary TB, 86.9% were new and 13.2% were retreatment. Wasting (i.e. body mass index of <18.5 kg/m^2^) was found in 38.5% of the patients and 63% presented with cough. Constitutional symptoms (fever, anorexia, night sweats and weight loss) were reported by 32.1%. Most patients (78.6%) presented with non-cavity lung parenchyma disease (infiltrates, nodules, masses) but 35.2% had cavity disease. Pleural disease was detected in 19.3% of patients. Positive smear microscopy and culture (irrespective of month of treatment) was found in 52.7% and 36.5% of patients respectively. Any drug resistance was detected in 21.1% of patients while multidrug resistance (MDR) TB defined as resistance to rifampicin and isoniazid was detected in 6.3% of patients. All MDR patients were new patients.

The prevalence of TB risk factors were as follows: HIV 41.4%, diabetes 5.4%, close contact 11.5%, family history 17.5%, smoking 26.37%, poverty 39.5%, overcrowding 57.3% and alcohol use 50.7%. Overcrowding increased smear positive rate, prevalence ratio 1.22, p = 0.09 but all the other studied risk factors did not affect clinical, radiological and mycobacteriological study patient characteristics.

**Conclusions:**

Among TB patients in Kampala, Uganda, there is high prevalence of the known TB risk factors. Targeting reducing their prevalence may lead to better TB control in the country. Tuberculosis, risk factors, Uganda.

## Background

Uganda is one of the 22 high tuberculosis (TB) burden countries (HBC) in the world [1]. From an estimated population of 35 million people with national HIV prevalence of 7.3%, 45,546 TB patients were diagnosed in the in the year 2010 of which 54% were HIV-infected [1-4]. Of these 56% were smear positive, 28% were smear negative and 11% had extra pulmonary TB [1].

Despite implementation of the WHO recommended directly observed therapy short course(DOTS)TB control strategy, the reductions in the incidence of TB have been minimal in HBC [5]. Because of this slow decline of TB incidence there is currently renewed interest in finding new TB control strategies. Focus has been on such strategies as adding to the current arsenal of TB drugs, finding a TB vaccine and designing shorter TB regimens. However, knowledge of what makes some persons develop TB and others not (risk factors) has potential of helping further to refocus the search for novel public health TB control strategies. Reported TB risk factors include HIV infection, male sex, co-morbidities such as diabetes, and family history of TB, absence of a Bacillus Calmette–Guérin (BCG) scar, smoking, alcohol use, single marital status, overcrowding, and poor socioeconomic status [6-9].

Infection with HIV has been the mostly associated risk factor for TB diseases in most HBC. [1] However, epidemiologic studies have found that not all TB cases can be attributed to HIV [10-13]. For example in Tanzania the population attributable fraction of TB due to HIV was 29% while in Nigeria it was reported as 40% [11,12]. These findings highlight the importance of other TB risk factors in perpetuating TB epidemics. Two of the risk factors that are increasingly being recognized as major drivers of the TB epidemic are tobacco smoking and diabetes [14-16]. Basu et al. through a mathematical model have shown that if current smoking trends continue, tobacco smoking will be responsible for 18 million TB cases and 40 million deaths in the next 40 years [17]. A recent meta-analysis has found a strong association (relative risk of 3.1) between TB and diabetes [18]. The association between TB, tobacco smoking and diabetes highlight the likely convergence of the emerging non communicable diseases (NCD) epidemic in developing countries and communicable diseases such as tuberculosis [19].

We conducted this study to determine the prevalence of known TB risk factors among TB patients diagnosed in Kampala district in Uganda and explore their effect on clinical, radiological and mycobacteriological TB disease presentation.

## Methods

### Design and setting

This was across sectional study of patients from selected government TB diagnostic and treatment units (DTUs) in Kampala district. Participating DTUs were: Kisenyi Health Center, Kiruddu Health Center, Kawaala Health Center, Kitebi Health Center, Kawempe Health Center, Kiswa Health Center and Mulago hospital. Kampala district was chosen because of its accessibility and large number of TB patients from all over the country (25% of total national TB notifications are from Kampala district) [20].

### Study participants

Participants were adult TB patients aged 18 years and above consecutively enrolled from participating DTUs during the period September 2012 through February 2013.

### Sample size calculation

Because the prevalence of most of the studied risk factors was not known we assumed a 50% prevalence rate. Targeting a precision of 0.05 and standard normal deviation at 95% confidence of 1.96 and allowing 10% non-response rate we estimated that 423 patients would be required to obtain precise estimates of the prevalence of the studied risk factors. This was not attained due to slow enrolment and an unexpected TB drugs stock out during the study period that affected enrolment of patients who were on continuation phase of TB treatment.

### Study procedures

Patients were interviewed using a structured questionnaire to collect demographic, socioeconomic and TB symptom data. All patients provided one sputum sample for mycobacteriological tests. At the study laboratory, smear microscopy was done by concentrated fluorescent microscopy method, after culture inoculation [21]. All samples were decontaminated for culture using standard operating procedures and all cultures were incubated for up to 8 weeks on two Lowenstein Jensen (LJ) media [22]. Culture results were communicated to the health facilities for patient management. A patient’s culture result was considered contaminated only if all LJ slants had non-mycobacterial growth, otherwise, positive result was taken as a single colony on solid media and AFB on Ziehl-Neelsen microscopy and/or capilia TB Neo™ positive, if doubtful colonies were observed. The absence of colonies on LJ medium after 8 weeks was reported as a negative result. Positive cultures underwent Drug Susceptibility Testing (DST) testing to determine if drug resistance was present. DST was performed by Automated Mycobacterial Growth Indicator tube method (MGIT960) as previously described [22].

Only patients at TB treatment initiation (month 0) underwent chest X-ray (CXR) examination and fasting blood sugar testing. Blood sugar was measured by Cobas intergra 400 version 3.4.4.0809 machine. All patients were HIV tested according to the ministry of health rapid HIV testing algorithm [23].

### Definition of variables

Tobacco smoking was defined as current or former use of at least 100 cigarettes in a life time [24]. Alcohol use was defined as history of drinking alcohol at least three times a week as previously used in other alcohol use studies in Uganda [24]. We defined poverty as a daily income of less than 1.25 USD/day [25] while overcrowding was defined as two or more adult persons who are not of opposite sex sharing a room [26].

Diagnosis of diabetes was made according to American Diabetes Association cut off ranges (<99 mg/dL, normal, 100-125 mg/dL, pre diabetes and ≥126 mg/dL, diabetes) [27]. We did not consider symptoms to make a diagnosis of diabetes. Abnormal CXR was defined as any abnormality on the CXR. Chronic cough was defined as cough lasting two or more weeks.

### Data analysis

Data were entered into an access database, cleaned and analyzed using epi-info software version 3.54. Descriptive statistics was used to summarize patients’ socio-demographic, clinical, radiological and mycobacteriological characteristics. Prevalence of different risk factors was calculated as a proportion of those with the risk factor divided by the total number of patients who had data for that risk factor. Proportions of key clinical (chronic cough), mycobacteriology (smear positivity) and radiological (abnormal CXR) were calculated and compared between the different risk factors studied using a *t*-test. A p value of <0.05 was considered to represent significant difference.

### Ethics consideration

All patients provided written informed consent. Ethical approval was obtained from the Makerere University School of Biomedical Science Research and Ethics committee and from the Uganda National Council of Science and Technology. Each patient was assigned a unique study number for identification. Patient’s names were not used on any study related documents. Study documents were kept under lock and key.

## Results

### Socio-demographic characteristics

A total of 365 TB patients were recruited during the study period. One hundred and forty one (38.6%) were at month zero, 84 (23.0%) were at month two, 64 (17.5%) were at month 5, 33 (9.0%) were at month six and 43 (11.8%) were at month eight of TB treatment.

Study patients characteristics are shown in Table 1. Male patients were 158 (43.3%), median age was 29 (IQR 28–30) and 296 (81.1%) were under the age 40 years.

**Table 1:** **Study patients’ characteristics**

**Characteristic**	**Level**	**No (%)**
**Demographic characteristics**		
Age	<40	296 (81.1%)
	41-65	68 (18.6%)
	>65	1 (0.3%)
Gender	Male	158 (43.3)
	Female	207 (56.7)
Occupation	Un/ self-employed	110 (64.0%)
	Employed	62 (36.0%)
Education	None	19 (5.2)
	Primary	110 (30.1)
	Secondary	39 (10.7)
	Tertiary	108 (29.6)
**Clinical characteristics**		
Site of TB (260)	Pulmonary	232 (89.2)
	Extrapulmonary	28 (10.8)
Treatment category	New	317(86.9)
	Retreatment	48(13.2)
BMI < 18.5 kg/m2 (n = 234)	<18.5	90 (38.5)
TB symptoms (n = 365)	Cough	230 (63.0)
	Constitutional symptoms	117 (32.1)
HIV infection (n = 365)	HIV infected	153(41.9)
**Radiological characteristics**		
CXR findings (n = 145)	Parenchymal (nodules, infiltrates, masses)	114 (78.6)
	Cavities	51 (35.2)
	Pleural (pleural effusion, pleural thickening)	28 (19.3)
	Adenopathy (hilar & mediastinal)	4 (2.8)
**Mycobacteriology characteristics**		
Smear	Negative	123 (47.3%)
	Positive	137 (52.7%)
Culture and drug sensitivity	Negative	165 (63.5%)
	Positive	95 (36.5%)
	Any drug resistance (INH, R, Z, E)	20 (21.1%)
	MDR	6 (6.3%)

Majority of the patients (89.2%) had pulmonary TB, 86.9% were new and 13.2% were retreatment. 38.5% were wasted (i.e. body mass index of <18.5 kg/m^2^) and 63% presented with cough. Constitutional symptoms (fever, anorexia, night sweats and weight loss) were reported by 32.1%.

Most (78.6%) presented with either infiltrates, nodules or masses, 35.2% cavities and 19.3% of the patients had TB disease presenting with pleural effusion and/or thickening.

Positive smear microscopy and culture (irrespective of month of treatment) was found in 52.7% and 36.5% of patients respectively. Of the 95 patients with 95 isolates any drug resistance was detected in 21.1% of patients while multidrug resistance (MDR) TB defined as resistance to rifampicin and isoniazid was detected in 6.3% of patients. All MDR patients were new.

### TB risk factors

The prevalence of each of the investigated TB risk factors is shown in Table 2. The prevalence of HIV infection was 41.4% (151 of 365) while that of diabetes was 5.4% (7 of 130). Although only 7 patients had blood sugars levels diagnostic of diabetes, another 12 patients (9.2%) had blood sugar levels diagnostic of pre-diabetes.

**Table 2:** **Prevalence of studied tuberculosis risk factors among study participants**

**Risk factor**	**Number with risk factor**	**% (95% CI)**
Income below poverty line (1.25 USD/day)	144	39.5 (34.4-44.7)
Overcrowding (>2 persons/room)	209	57.3 (52.0-62.4)
Former smoker	96	26.3 (21.9-31.1)
Current smoker	5	1.4 (0.4-3.2)
Alcohol use	185	50.7 (45.4-55.9)
HIV infection	151	41.4 (36.3-46.6)
TB contact	42	11.5 (8.4-15.2)
Family history of TB	64	17.5 (13.8-21.8)
Diabetes	7	5.4 (3.1-6.7)

Current smoking was 1.4% (5 of 365) but history of smoking was reported by at 26.3% (96 of 365) of the patients and 52.6% of former smokers reported to have quit in the past one year.

Poverty was found in 39.5%(144 of 365) while overcrowding was reported by 57.3% (209 of 365) patients. One hundred and eighty five patients (50.7%) reported alcohol use.

More patients (57.6%) from overcrowded homes were smear positive compared to those from homes without overcrowding (47.1%), prevalence ratio (PR) of 1.22, p = 0.09. We however found no other significant differences in the clinical, radiological and mycobacteriological TB disease presentation between other risk factors (Table 3).

**Table 3:** **Tuberculosis risk factors and selected tuberculosis disease presentation**

**Independent variables**	**Smear positive**	**PR (SE), p value**	**Abnormal chest x-ray**	**PR (SE), p value**	**Chronic cough**	**PR (SE), p value**
	**N (%)**		**N (%)**		**N (%)**	
**Poverty**						
Yes	17 (53.1)	1.10 (0.21)	12 (37.5)	1.07 (0.28)	15 (46.9)	0.81 (0.16
No	61 (48.4)	0.625	44 (34.9)	0.783	73 (57.9)	0.296
**Overcrowding**						
Yes	80 (57.6)	1.22 (0.15)	54 (38.9)	1.04 (0.17)	80 (57.6)	1.09 (0.12)
No	57 (47.1)	0.097	45 (37.2)	0.784	64 (52.9)	0.453
**Smoking**						
Yes	45 (58.4)	1.16 (0.14)	27 (35.1)	0.89 (0.16)	40 (52.0)	0.92 (0.12)
No	92 (50.6)	0.230	72 (39.6)	0.503	103 (56.6)	0.501
**Alcohol use**						
Yes	77 (55.8)	1.13 (0.14)	50 (36.2)	0.90 (0.14)	78 (56.5)	1.04 (0.12)
No	60 (49.2)	0.290	49 (40.2)	0.514	66 (54.1)	0.695
**HIV infection**						
Positive	128 (52.9)	1.1 (0.26)	90 (37.2)	0.74 (0.19)	137 (56.6)	1.46 (0.44)
Negative	9 (50.0)	0.817	9 (50.0)	0.237	7 (38.9)	0.212

PR = Prevalence Ratio, SE = Standard Error.

## Discussion

In this cross sectional study among TB patients under care in Kampala, Uganda we have established the prevalence of key known TB risk factors as follows: HIV infection 41.4%, alcohol use 50.7%, poverty 39.5%, smoking 26.3%, family history of TB 17.5%, TB contact 11.5% and diabetes 5.4%.

High prevalence of HIV among TB patients is due to HIV associated immune suppression which increases risk for TB through reactivation of latent TB disease and increased proportion of persons who progress from TB infection to disease. The annual risk of TB among HIV patients is 10%/year compared to the 10% life time risk among HIV negative persons [28]. The prevalence of HIV found in this study is about 6 times (41.4 vs.7.3) that in the general population. The prevalence is however lower than the over 50% prevalence previously reported [1]. This difference could be because of the small sample size and small number of recruitment sites. However, a study investigating prevalence of MDR-TB by Lukoye D et al. reported lower HIV prevalence of 30.5% compared to the current study [29].

The association between tobacco smoking and TB is well described [15,17,30]. The prevalence we report in this study does not differ significantly from those reported in other studies in Africa [31] but differs from those in other regions [32]. For example a study in South Africa reported a prevalence of 21.8% while a study in Malaysia reported a prevalence of 40.3% [33]. These differences could be attributed to background smoking prevalence in the general population. The prevalence of background tobacco smoking in Uganda population is limited because population based surveys have not been conducted. A review conducted by Nturibi EM et al. identified three studies on tobacco smoking prevalence in Uganda [34]. The prevalence in these studies ranged from 5.3% to 21.9% [35]. It must be noted all the three studies included mainly young people in schools. An unpublished fact sheet from the recently concluded GATS tobacco use survey in Uganda reported a prevalence of 12% in the general population (http://www.afro.who.int/en/uganda/press-materials/item/6713-uganda-releases-results-of-the-global-adult-tobacco-survey-2013.html). We also noted that the prevalence of current smoking was only 1.37% but on further evaluation it was found that about half of the former smokers had actually quit in the past year. This could be due to fact that most patients stopped smoking when they developed TB respiratory symptoms interpreting them as due to smoking.

Previous studies have documented a strong association of diabetes and TB [18,36]. A recent meta-analysis has found a strong association (relative risk of 3.1) between TB and diabetes [18]. The prevalence reported in this study is lower than that reported in a recent study among hospitalized TB patients in Mulago Hospital, Kampala [34]. This difference could be due to the fact that stress hyperglycemia in the hospitalized patients may have contributed to more patients being diagnosed with diabetes. The study also used random blood sugar instead of the recommended fasting blood sugar. In addition 12 patients met the criteria of pre-diabetes. Taking the pre-diabetes and the diabetes together gives a prevalence of 10.8% comparable to what was found in the Mulago hospital study.

We investigated the number of patients whose daily incomes were below the poverty line and how many were living in overcrowded homes. The prevalence of poverty we found (39.5%) was slightly higher than the national average of 38% [37]. We note that this study was conducted in the capital city of Uganda. Therefore patients’ incomes may be higher than 1.25 USD/day designated by the World Bank. In the 2012–2013 when the study was conducted 1.25 USD/day could barely afford a simple lunch meal. In essence this cut off may grossly underestimate the poverty levels of the populace especially those in urban areas. Uganda with 53% of the population living in overcrowded homes is number 14 on the list of the 20 countries with most overcrowded homes in the world [26]. Overcrowding has been previously documented as a strong risk for TB [38]. In this study we found that 57.3% of the study participants were living in overcrowded homes. This is higher than the national average. The national figures do not take into account the TB burden in the community. The study area (Kampala) has the highest prevalence of TB in the country. One in four TB cases notified in Uganda are from Kampala [20]. Therefore overcrowding may represent a greater risk of TB than in areas where the TB is much lower.

Close contact is another well described risk factor for TB [39-44]. Close contact was estimated to account for 9-13% of the TB cases in Malawi [44]. In a systematic review by Fox GJ et al. the prevalence of active TB and latent TB infection among TB contacts was 3.1% and 51.5% respectively [40]. A prevalence of 3.1 represents a major increase in TB prevalence compared to the general population. For example in Uganda TB prevalence is 175/100,000 population (0.02%) [1]. It follows therefore that TB prevalence among contacts is very high [1]. The odds ratio of having a family member with TB and developing TB is estimated at 13.4 further highlighting the importance of close TB contact and TB risk [6]. This factor is not well studied among patients in most TB programs. We contend that a prevalence of family history of TB of 17.5 is high and need further investigations. This would mean that beyond close contacts health workers would elicit family history of TB, the presence of which would refine targeting of TB screening strategies.

We also investigated the prevalence of alcohol use among TB patients. The association of TB and alcohol is well described in the literature [45,46]. It can be hypothesized that it is due to the close contact that takes place in bars as opposed to other social gatherings. Lanoroth K et al. conducted a systematic review in which they established that alcohol use was independently associated with TB (RR 2.94). More than 50% of the TB patients in our study consumed alcohol. Although we investigated the effect of the studied risk factors on TB disease presentation we did not find significant influence of the risk factors on disease presentation. Marginal influence was observed between overcrowding and smear positive rate. This could be due to the fact that our study was not powered to detect these differences. Secondly we enrolled patients at different stages of treatment which could have affected the prevalence of the outcomes variables.

Lastly we found an MDR rate of 6.3% and all were from new patients. This prevalence is much higher than that found in the national drug resistance survey conducted in 2010 (1.4%) and that found in the Kampala drug resistance survey conducted in 2008 (1.1%) [29,47]. This difference could be due to smaller sample size since we had only 95 culture positive patients. It could also be sampling bias because we included only government DTUs and enrolled patients during one reporting quarter. We also included TB patients irrespective of duration on TB treatment. Therefore some new patients could have started developing drug resistance during the time they were on TB treatment. We also noted that during the study period there were drug stock outs at study clinics. This could have further facilitated emergence of drug resistance among study patients. These shortcomings not withstanding these results emphasize the role of surveillance of drug resistance. A follow up survey or analysis of routinely collected data can help clarify if there could be increase in drug resistance in the country.

This study had some limitations. Firstly the patients were recruited over one quarter of the reporting year. It is possible that there may be differences in the patients who reported to the health facilities during other times. We defined overcrowding using alternative method of number of persons per room. This method does not take into account the size of the room. This was done because we could not obtain accurate room sizes in order to calculate the living area per person. Patient incomes were also self –reported. We used daily income as a measure of poverty. However we recognize that a more extensive investigation of poverty parameters would provide different estimates. We did not take into consideration symptoms while making a diagnosis of diabetes as recommended. Only 86% of the estimated sample size was achieved under convenient sampling, future studies with bigger sample size are recommended. Nevertheless, much as this study is more qualitative than quantitative, it is one of few studies specifically conducted to investigate the prevalence of TB risk factors among TB patients. It has generated data that should guide more detailed, possibly better designed quantitative studies.

## Conclusions

Among TB patients in Kampala, Uganda, there is high prevalence of the known TB risk factors. Targeting reducing their prevalence may lead to better TB control in the country.

## Abbreviations

ART: Antiretroviral therapy
CD4: Cluster of differentiation 4
CI: Confidence interval
CXR: Chest X-ray
EDTA: Ethylene diamine tetra acetic acid
DST: Drug susceptibility testing
DTU: Diagnostic and treatment unit
HBC: High burden country
HIV: Human Immunodeficiency Virus
IQR: Inter quartile range
MDRTB: Multidrug resistant TB
MGIT: Mycobacterial growth indicator tube
NCD: Non communicable disease
RR: Relative risk
TB: Tuberculosis
USD: United States Dollar
